# Blood Pressure Tracking From Childhood to Adulthood

**DOI:** 10.3389/fped.2021.785356

**Published:** 2021-11-15

**Authors:** Tatsuhiko Azegami, Keiko Uchida, Mitsuaki Tokumura, Masaaki Mori

**Affiliations:** Keio University Health Center, Yokohama, Japan

**Keywords:** blood pressure, tracking, cardiovascular disease, hypertension, prediction

## Abstract

Hypertension is the most common non-communicable disease among adults and is the most important modifiable risk factor for premature cardiovascular disease. The increasing worldwide burden of hypertension is a major global health issue. Early prevention with lifestyle modification or pharmaceutical treatment reduces the incidence of hypertension and the risk of subsequent cardiovascular disease. Therefore, identification of young persons at risk for hypertension has the obvious benefit of providing a chance for early intervention. Previous studies have demonstrated the positive association of elevated childhood blood pressure with hypertension in adulthood. Accumulated evidence also indicates the possibility that elevated pediatric blood pressure is associated with increased risk of future cardiovascular disease. In this article, we review the tracking of blood pressure from childhood to adulthood and emphasize the importance of pediatric blood pressure monitoring and control for predicting and preventing adult hypertension and cardiovascular disease.

## Introduction

According to the World Health Organization, an estimated 1.28 billion adults worldwide have hypertension (1). The global prevalence of hypertension among adults has doubled in the last 30 years, with most of the increase occurring in low-income and middle-income regions (2). In addition to having a high prevalence, hypertension is recognized to be the most important modifiable risk factor for premature cardiovascular diseases (3). Each 20-mmHg increase in systolic blood pressure (BP) is associated with a more than 2-fold increase in death rates from stroke and ischemic heart disease among middle-aged persons. Because stroke and heart disease contribute to the impairment of activities of daily living (ADL) (4, 5), hypertension in middle age is associated with future decline in ADL (6). Furthermore, hypertensive persons spend nearly $2,000 more annually on healthcare than individuals without hypertension (7), and suboptimal blood pressure accounts for 10% of the world's overall healthcare expenditure (8). Therefore, control of hypertension is extremely important, not only for preventing life-threatening complications and subsequent impaired ADL but also as a cost-containment strategy for both people and social healthcare systems.

To combat the global burden of hypertension, a population-based comprehensive approach to lowering blood pressure may be an attractive strategy because although it might achieve only a small reduction in blood pressure in each person, it has the potential to decrease the risk of cardiovascular disease (CVD) in a large number of people (9). Moreover, a targeted approach is an effective strategy for preventing hypertension in persons at high risk of developing hypertension or CVD (10). In a targeted approach, in general, the earlier that reversible risk factors can be corrected, the more effectively future CVD can be prevented. Therefore, early identification of hypertension and intervention in a high-risk population will help to overcome hypertension and CVD.

Childhood prediction models can help identify persons at risk of developing adulthood hypertension. Accumulated evidence points to associations of childhood health data with future BP status (11). For example, being overweight and having elevated serum uric acid levels in childhood are potential predictors of future hypertension (12, 13). Childhood BP is well-known to be the strongest predictor of future adult BP. In this review, we shed light on BP tracking from childhood to adulthood and discuss the usefulness of childhood BP as a predictor of adult BP.

## Predictors of Adult Hypertension

High BP is a major risk factor for death and a large contributor to global disability-adjusted life years (14). There is a causal association of high BP with cardiovascular events among not only the elderly but also young adults (15). In a longitudinal cohort study that followed young adults aged 18–30 years for a median of 18.8 years, the adjusted hazard ratio for CVD events for elevated BP (untreated systolic BP 120–129 mmHg and diastolic BP <80 mmHg) in young adults vs. normal BP (untreated systolic BP <120 mmHg and diastolic BP <80 mmHg) was 1.67 (95% confidence interval, 1.01–2.77) (15). Therefore, early identification of elevated BP may be a promising strategy for intervention in high-risk populations.

**Figure 1:**
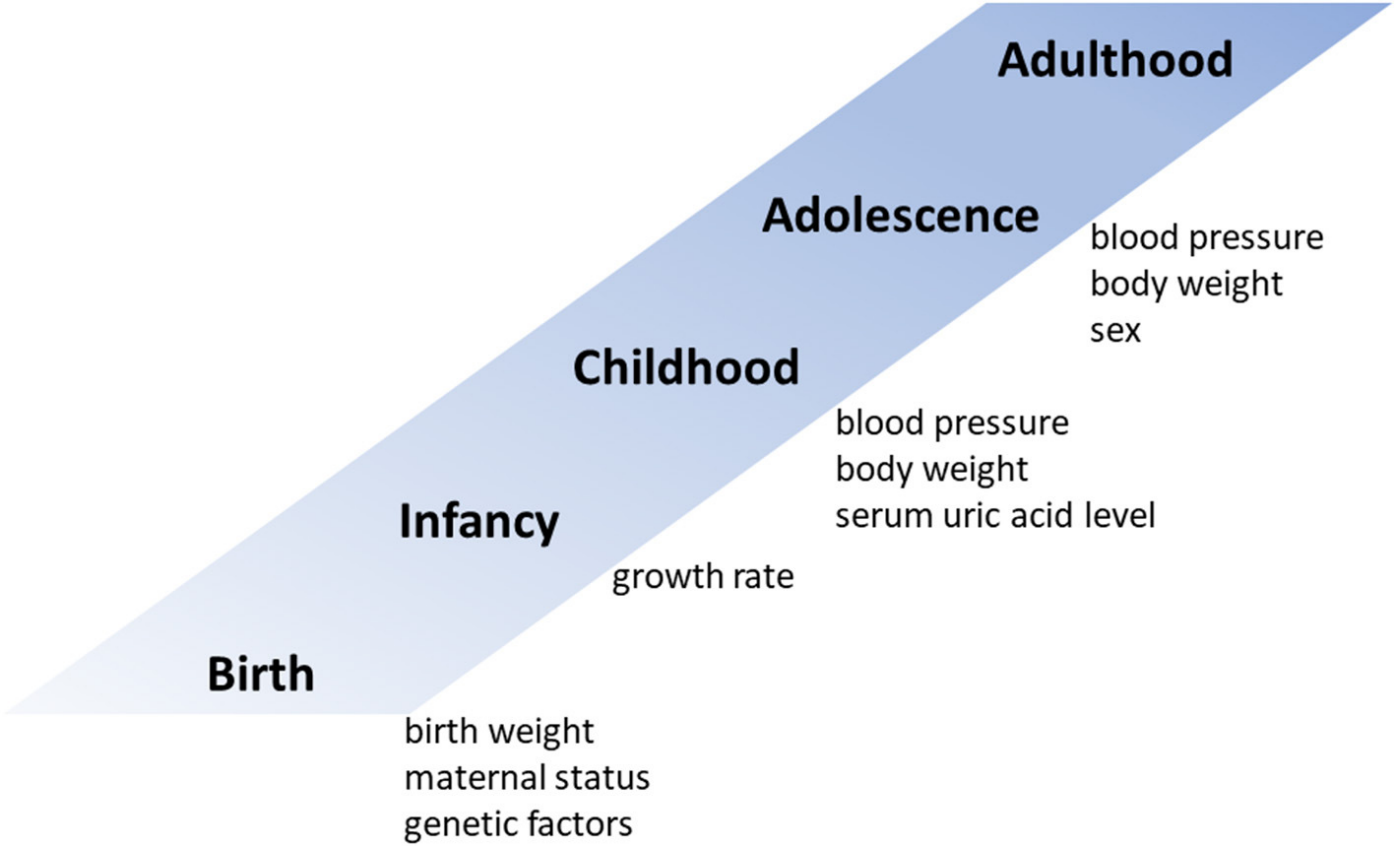
Predictors of blood pressure in adulthood.

Both genetic and environmental risk factors contribute to elevated BP levels (Figure 1). Family (parental) history of hypertension is a strong hereditary risk factor for future hypertension (12). One monozygotic twin study estimated the heritability of hypertension to be 61% (16). Although their contribution to future blood pressure levels is not very high, several studies have shown that genetic polymorphisms such as angiotensin converting enzyme (*ACE*) and angiotensinogen (*AGT*) are associated with future blood pressure levels (17–19). As unmodifiable predictors of adult hypertension, low birth weight and maternal gestational diabetes are also associated with hypertension in offspring (20, 21). In addition, adults who experience catch-up growth during early childhood have higher BPs than those without accelerated weight gain during this period (22).

In addition to unmodifiable predictors, some modifiable childhood factors are associated with future BP and may have the potential to predict adult hypertension. Second to childhood hypertension, obesity during childhood is the most frequently reported risk factor for hypertension later in life (12, 23–26). In a simple regression model, compared with children with a body mass index (BMI) in the <50th percentile, boys in the ≥85th percentile were 13.2 times more likely and girls in the ≥85 percentile were 48.2 times more likely to become hypertensive during young adulthood (25). However, in contrast, a multivariate regression analysis indicated that height, weight, and BMI in childhood did not predict young adult blood pressure after adjustment for childhood blood pressure; moreover, not childhood height and weight but their change from childhood to adulthood was a strong predictor of young adult blood pressure (24). In a multivariable logistic regression analysis conducted after these studies, childhood overweight or obesity was reported to be an independent predictor of young adult hypertension even after adjustment for childhood hypertension; children with overweight or obesity had an odds ratio of developing future hypertension of 1.65 (95% confidence interval, 1.16–2.34) compared with non-overweight children (12).

Childhood serum uric acid level is another potential predictor of young adult blood pressure (13). In the Bogalusa Heart Study, adults with hypertension had higher childhood serum uric acid levels than normotensives (5.12 vs. 4.30 mg/dL), and serum uric acid level during childhood and its change from childhood to adulthood were independent predictors of adult BP, as identified in a multivariate regression model (13). In our previous study, a multivariable logistic regression analysis revealed that a model including serum uric acid level in addition to systolic BP and body weight had the highest predictive power for young adult BP (11).

In addition, some disease states in childhood may be associated with elevated BP in adulthood. In a recent 10-year prospective follow-up study, obstructive sleep apnea in childhood was associated with a higher nocturnal systolic BP of 6.5-mmHg and reduced nocturnal dipping of systolic BP at follow-up, indicating that it was a risk factor for future hypertension independent on BMI (relative risk, 2.5; 95% confidence interval, 1.2–5.3) (27). Kidney injury and cancer were also associated with the risk of subsequent hypertension, with adjusted odds reported to be 2.2- and 2.6-fold, respectively (28, 29).

## Association Between Childhood and Adulthood Blood Pressure

BP is a continuous variable, and it is difficult to set a universal cut-off value that determines whether a BP above that value is harmful to all persons. Therefore, adult BP guidelines determine hypertension cut-off values on the basis of the magnitude of the risk of cardiovascular disease (30–32). However, children and adolescents are at quite low risk of having cardiovascular disease, and their BPs are much lower than those in adults. Therefore, given the lack of outcome data, the definition of hypertension in children and adolescents is based on the distribution of BP in healthy subjects (33). For example, according to the recent American Academy of Pediatrics guideline, childhood BP levels are interpreted on the basis of sex, age and height, and hypertension is defined as BP ≥95th percentile (33). Establishing a predictive model for BP trajectories and further investigating the relationship of childhood BP with adult hypertension and subsequent cardiovascular disease will yield valuable evidence to improve and strengthen the future categorization of pediatric BP.

Theodore et al. tackled this clinical issue and provided some helpful insights (34). In a longitudinal study, they obtained BP data for 975 subjects at ages 7, 11, 18, 26, 32, and 38 years, and they identified four BP trajectory groups across these ages (34). They found that participants who developed high BP as adults tended to have elevated BP in childhood. Moreover, subjects in the high-BP trajectory group were more likely to have cardiovascular risk factors such as high cholesterol levels at age 38 years (34). Similarly, Hao et al. identified three BP trajectory groups during a 23-year period in 683 subjects followed from childhood to young adulthood (35). Compared with subjects started with a low level in and maintained a low increase in systolic BP, subjects started with a high level in and had a fast increase in systolic BP had greater intima–media thickness (IMT) and a higher left ventricular mass index in adulthood, suggesting that a high rate of increase in BP from childhood to young adulthood was associated with increased subclinical cardiovascular risk (35). In both studies, a common factor for categorization in the high-BP trajectory group was a high BMI during childhood (34, 35).

Because it is difficult to monitor BP over a long period of time, only a few studies have tracked BP from childhood to middle age (36, 37). One study measured BP annually and examined the correlation of childhood and adolescent BPs with BP in middle age. It revealed that juvenile BP measured during the early school years and in early puberty is a strong predictor of BP at age 50 years, especially in men (36). Similarly, middle-aged adults with hypertension are reported to have had higher systolic BP, diastolic BP, BMI, and serum triglyceride levels during childhood (ages 8–11 years) than have normotensive adults (37). Surprisingly, the prevalence of adulthood hypertension (at a mean age of 46.7 years) rises from 19% for subjects who were persistently normotensive in both childhood and adolescence to 80% for individuals with persistently elevated BP in both childhood and adolescence (37). As is generally known, both systolic and diastolic BP increase with age. However, interestingly, the characteristics of the BP slope from childhood to middle age vary by race and sex (38). BP from ages 5–14 years does not vary by race and sex, but BP growth curves diverge from age 15 years, with the slopes of both systolic and diastolic BP being steeper in males than in females before age 45 years in African Americans and before age 50 years in Caucasian Americans (38).

In our previous study we, too, found a sex-associated difference in adolescent BP slope. BP in boys increased from 105.7/57.5 mmHg at age 12 or 13 years to 117.8/63.6 after a mean follow-up of 8.6 years, whereas BP in girls did not change during the same period (from 105.2/59.3 mmHg to 105.2/59.6 mmHg) (11). This difference in BP increase during adolescence between males and females may be at least partially caused by differences in sex hormones. In an experiment in female adolescent mice, ovariectomy led to an increase in BP and estrogen supplementation partially normalized increased BP (39).

Although it was conducted over a decade ago, a meta-regression analysis on BP tracking from childhood to adulthood was published in 2008 (40). The meta-regression analysis included 50 cohort studies followed for 0.5–47 years and examined the BP tracking correlation coefficient which measured the degree of correlation between 2 repeated observations over time (40). Interestingly, the BP tracking correlation coefficient became smaller with longer follow-up and larger with age at the baseline (40). Compared to studies with baseline ages <5 years, study with baseline age of 5 years or older showed strong tracking correlations that were independent of follow-up period (0.18 vs. 0.40 for SBP and 0.09 vs. 0.29 for DBP) (40), suggesting that childhood BP values after age 5 years may closely influence future BP values.

## Association Between Childhood Blood Pressure and Future Cardiovascular Disease

As mentioned above, both modifiable and unmodifiable childhood factors are associated with future adult hypertension. Elevated childhood BP, especially, has the potential to be a strong and promising predictor of adult hypertension. However, sufficient evidence to confirm the direct association between elevated childhood BP and future CVD has still not been accumulated, because it is extremely difficult to conduct prospective studies that require long-term follow-up for nearly half a century. Few studies have therefore examined the associations between elevated BP in young people and subsequent CVD and CVD-related mortality.

In a Swedish longitudinal cohort study, the direct association of BP in late adolescent males (ages 18–20 years) with subsequent coronary heart disease (CHD) or stroke before age 55 years was examined (41). In multivariate analyses, adjusted hazard ratios for CHD increased gradually for systolic BPs exceeding 115 mmHg and diastolic BPs exceeding 75 mmHg. In contrast, elevated systolic BP in late adolescence did not significantly increase the hazard ratio for stroke, but a diastolic BP of ≥85 mmHg did increase it. A subsequent Swedish study also revealed that not systolic BP but a diastolic BP of ≥71 mmHg during late adolescence was an independent risk factor for future CVD-related mortality (42). The reasons why adolescent diastolic BP is superior to systolic BP as a predictor of subsequent stroke and death have not been fully elucidated. However, the findings that the impact of diastolic BP on the risk of new-onset hypertension is greater than that of systolic BP in young adults (43) and that diastolic hypertension is associated with obesity-related hypertension (44) may contribute at least partially to the predictive impact of diastolic BP.

To compensate for the lack of sufficient findings of an association between BP in the young and subsequent adult CVD, we may be able to conduct retrospective analyses of longitudinal cohort studies to evaluate surrogate markers for CVD or organ damage, including left ventricular hypertrophy (LVH), IMT, and arterial stiffness, as alternatives to hard endpoints such as cardiovascular death. For example, one longitudinal cohort study from childhood to adulthood suggested that the slope of systolic BP during childhood and adolescence was positively associated with adult LVH, even after adjustment for adult BP; moreover, the odds ratio for the association of BP slope with adult LVH increased with age in adolescents (45). An association between childhood BP and future adult LVH has been found in other studies: high BP in childhood is associated with high left ventricular mass and LVH after adjustment for race, sex, and age (46), and elevated childhood BP is associated with increased risk of adulthood hypertension, arterial stiffness, and LVH (47).

The positive association between childhood BP and adult IMT was also clearly indicated in a recent longitudinal cohort study (48). Per standard deviation change in each predictor, childhood systolic BP had the highest age- and sex-adjusted odds ratio for ≥90th percentile of adult carotid IMT (1.24; 95% confidence interval, 1.13–1.37) (48). The cut-off values for elevated systolic BP in children and adolescents, based on an association with the development of increased carotid IMT in adulthood, were 123 mmHg for boys and 115 mmHg for girls at ages 13–18 years (48). Brachial-ankle pulse wave velocity (baPWV) has been used as a simple method of evaluating arterial stiffness and its level is positively associated with increase in the risk of CVD (49). In a longitudinal cohort study of 835 subjects followed for an average of 26.5 years, childhood systolic BP was found to be an independent predictor of baPWV in adulthood (50).

## Early Intervention for High Blood Pressure in Childhood

A few recent studies, as outlined above, have shown that elevated childhood BP is closely associated with adult hypertension and the development of CVD. However, insufficient evidence has been accumulated as to whether early intervention for childhood BP can prevent the development of adult hypertension and CVD.

Weight control in childhood and adolescence may be a promising approach to preventing adult hypertension. Although they did not directly confirm the preventive effect of weight reduction on subsequent hypertension, Hou et al. demonstrated that, compared with normal weight in both childhood (ages 6–17 years) and young adulthood (ages 18–37 years), for overweight in both childhood and adulthood the relative risk of adult hypertension was 3.79 (95% confidence interval, 2.49–5.64). In contrast, for overweight in childhood and normal weight in adulthood the relative risk was 1.05 (95% confidence interval, 0.33–3.40) (51), suggesting the importance of weight control during childhood for preventing adult hypertension. Although the effect decreases over time, it has also been shown that higher physical fitness in childhood may reduce subsequent raised BP (52). In addition, if complications such as obstructive sleep apnea are present in childhood, their treatment may also prevent the development of hypertension in the future (53).

Salt intake reduction is another potential approach to inhibiting the future development of hypertension. Although, to our knowledge, no prospective human clinical trial has evaluated the long-term effect of salt reduction during childhood on adult BP, a study in rats found that dietary sodium restriction during adolescence attenuated the development of adult hypertension (54). Other non-pharmacological interventions, such as physical training and the DASH (Dietary Approaches to Stop Hypertension) diet, have been shown to reduce childhood BP in randomized controlled trials (55–57), but their preventive effect on future hypertension has not yet been elucidated.

Pharmacological interventions also reduce BP in hypertensive children (58). However, their long-term effects—especially their preventive effect on adult hypertension—are uncertain. The renin–angiotensin system is involved in the development of childhood hypertension, and inhibition of angiotensin II by the receptor blocker candesartan cilexetil reduced blood pressure by 8.6–11.2/4.8–8.0 mmHg in hypertensive children aged 6–17 years (59). Moreover, high plasma angiotensin II and aldosterone levels are associated with LVH in hypertensive children (60). These findings suggest the importance of early inhibition of the renin–angiotensin system in childhood hypertension. Although only an animal study, one investigation in prepubescent rats showed that temporary inhibition of the renin–angiotensin system suppressed the subsequent development of hypertensive kidney injury (61, 62).

## Future Prospects

This review reaffirmed that childhood BP is an important factor in predicting future BP level. However, when considering childhood BP from the perspective of tracking, there are several issues that need to be considered in the future. The first question is where to set the BP threshold in children. It is robust that BP in childhood correlates with BP levels in later adulthood, but where should we set the cut-off value for intervention? The optimal threshold is considered to be the BP level in childhood, which is strongly associated with high risk of future hypertension and cardiovascular events in adulthood. Therefore, future studies are needed to prospectively track BP in childhood over a long period of time and investigate the relationship with hypertension and cardiovascular events in adulthood. Second, it should be recognized that pediatric hypertensives are a minor population and that a targeted approach targeting only those at high risk is insufficient to combat global burden hypertension and needs to be combined with a population approach. Third, there is no direct scientific evidence that early intervention (non-pharmacological and pharmacological) in pediatric hypertensives reduces the incidence of future hypertension or cardiovascular events. Therefore, in the future, prospective intervention trials for pediatric hypertension should be conducted to investigate the long-term effects.

## Conclusion

Both genetic and environmental factors are associated with the development of adult hypertension. Accumulated evidence suggests that a number of childhood health factors are good predictors of subsequent BP status. Therefore, we believe that childhood is an important period in the development of future hypertension and that early identification of children at high risk for adult hypertension and CVD, as well as early non-pharmacological and pharmacological intervention, is an attractive challenge for combating the global burden of hypertension. However, it should be noted that there are only few persons with elevated BPs during childhood and that a population-based comprehensive approach to lowering childhood blood pressure also has the potential to overcome adult hypertension and CVD.

Childhood BP is positively associated with adult BP, and this association can be tracked from childhood through to adulthood. Some factors, including race and sex, affect BP slope, but it remains uncertain which modifiable factors increase BP slope and whether interventions during childhood can reduce this slope. In addition, there is little evidence to directly confirm the causal association of elevated childhood BP with subsequent adult CVD. In future, if a gradual and progressive association is found between elevated childhood BP and increased risk of adult CVD, childhood BP will be categorized on the basis of not within-population distribution but estimated CVD risk.

## Author Contributions

TA contributed to design, literature review, and wrote the manuscript. KU, MT, and MM reviewed the manuscript. All authors contributed to the article and provided final approval for submission.

## Funding

This work was supported by the Keio University Academic Development Funds for Joint Research.

## Conflict of Interest

The authors declare that the research was conducted in the absence of any commercial or financial relationships that could be construed as a potential conflict of interest.

## Publisher's Note

All claims expressed in this article are solely those of the authors and do not necessarily represent those of their affiliated organizations, or those of the publisher, the editors and the reviewers. Any product that may be evaluated in this article, or claim that may be made by its manufacturer, is not guaranteed or endorsed by the publisher.
